# Significance of Testing Anti-Thyroid Autoantibodies in Patients with Deranged Thyroid Profile

**DOI:** 10.1155/2018/9610497

**Published:** 2018-04-11

**Authors:** Hamid Nawaz Tipu, Dawood Ahmed, Muhammad Mukarram Bashir, Naveed Asif

**Affiliations:** ^1^Immunology Department, Armed Forces Institute of Pathology, Rawalpindi, Pakistan; ^2^Chemical Pathology Department, Armed Forces Institute of Pathology, Rawalpindi, Pakistan

## Abstract

**Background:**

We hypothesized that anti-thyroid antibodies are more often positive in individuals with deranged thyroid profile.

**Methods:**

This prospective cohort was done in Immunology Department, Armed Forces Institute of Pathology, Rawalpindi, Pakistan, from Jan 2017 to Oct 2017. All the samples that were referred to us for testing anti-thyroid antibodies (anti-TPO or anti-TG antibodies) and thyroid profile were included in the study. There were no exclusion criteria. Tests for anti-thyroid antibodies were performed by ELISA and thyroid profile by chemiluminescence. SPSS 23.0 was used for statistical analysis.

**Results:**

Over a course of a ten-month study period, we received a total of 316 serum samples for anti-TPO/TG antibodies along with thyroid profile testing (TSH). These included 115 males (36.4%) and 201 females (63.6%). Their age ranged from 3 to 89 years (mean ± SD, 42.22 ± 18.09). Anti-TPO antibodies were more often positive when TSH was deranged (*p* value 0.001). Anti-TPO antibodies are more often raised in females, in terms of both prevalence (*p* 0.001) and mean rank (*p* 0.002).

**Conclusion:**

As anti-thyroid antibodies are more often present when TSH is deranged, such individuals should be screened for anti-thyroid antibodies. This importance of screening is compounded by the fact that anti-thyroid antibodies may be positive in a significant percentage of elderly people.

## 1. Introduction

Autoimmune thyroid disorders (AITDs) are a diverse group of organ-specific autoimmune diseases, the most common of which include Hashimoto's thyroiditis and Graves' disease [1]. Several predisposing genetic loci including CTLA4, HLA, and IL2RA have been identified and certain environmental factors like radioiodine treatment, iodine deficiency, and cigarette smoking have been implicated in pathogenesis of AITDs [2, 3]. Although AITDs occur in only 1% of population, subclinical and focal thyroiditis and circulating anti-thyroid antibodies may be found in 15% of euthyroid subjects [4]. Anti-thyroid peroxidase (TPO) antibodies arise against a transmembrane protein of thyrocytes involved in thyroid hormone synthesis. Anti-thyroglobulin (TG) antibodies are against thyroglobulin, a thyroid hormone precursor [1]. Anti-TPO antibodies (formerly known as anti-thyroid microsomal antibodies) and anti-TG antibodies are considered diagnostic of AITDs because these are present in over 90% cases of Hashimoto's thyroiditis and over 80% cases of Graves' disease [1].

Anti-TPO and anti-TG antibodies are related to levels of thyroid stimulating hormone (TSH) and both alone or in combination have been used to predict development of hypo-/hyperthyroidism. It has been determined in different studies that altered levels of anti-thyroid antibodies and TSH in euthyroid subjects have been associated with development of hypothyroidism in future [5, 6]. In fact, anti-thyroid antibodies are involved in pathogenesis of autoimmune thyroiditis through complement-dependent cytotoxicity [7]; hence, their appearance may actually precede development of overt thyroid disease or deranged thyroid function tests by several years [8]. Since anti-thyroid antibodies have been detected in healthy individuals especially females [9], follow-up thyroid profile testing in anti-thyroid antibody positive individuals is very important for making timely diagnosis.

The use of biomarkers to predict susceptibility and outcome in thyroid autoimmunity has steadily increased over time. Thyroid genetic susceptibility testing along with thyroid autoantibodies is highly predictive of later thyroid autoimmunity and thyroid dysfunction [10]. In Pakistan, the prevalence of both hyperthyroidism and hypothyroidism is approximately 4-5% [11]. Different studies have examined anti-thyroid antibodies in different subgroups of patients like in SLE (54.76%) [12], hepatitis C patients (anti-TPO 26.8%) [13], and chronic urticaria patients (anti-TPO 57.4% and anti-TG 42.6%) [14]. However, prevalence of anti-thyroid antibodies among general population is unknown. And so is its relation with thyroid profile (TSH, T4, and T3). Determining association of anti-thyroid antibodies with thyroid profile testing could identify such group of patients who have deranged thyroid profile and subsequently also need screening for thyroid autoantibodies to rule out underlying autoimmune process. Keeping in view, we hypothesized that anti-thyroid antibodies are more often positive in patients with deranged thyroid profile.

## 2. Patients and Methods

This prospective cohort study was carried out at Immunology Department of Armed Forces Institute of Pathology (AFIP), Rawalpindi, Pakistan, from January 2017 to October 2017, after formal approval of institutional ethical committee. Sampling was done through nonprobability consecutive sampling. All the samples that were referred to us for testing anti-thyroid antibodies (anti-TPO or anti-TG antibodies) and thyroid profile were included in the study. There were no exclusion criteria. Anti-TPO and anti-TG antibodies were both performed by sandwich ELISA using Spinreact, Spain, and Adaltis, Italy, kits, respectively, according to manufacturer's recommendations. Cut-off for anti-TPO antibodies was <20 AU/ml while for anti-TG antibodies it was 125 IU/ml, as recommended by manufacturers. TSH was performed by 3rd-generation assay employing anti-FITC monoclonal antibody (mAb) for chemiluminescence detection by ADVIA instrument. Free T4 was performed on random access ADVIA CENTAUR XP immunoassay system by direct chemiluminescence. Range for TSH was 0.4–4.5 mIU/ml and for T4 was 8.0–21.0 pmol/l, as recommended by manufacturers. Results were entered in Statistical Package for Social Sciences (SPSS) version 23.0. Chi square test was applied to determine statistical significance for string variables, that is, gender versus derangement of anti-TPO, anti-TG, TSH, and free T4. Ranges outside the normal values (above or below) were considered as deranged values. Mann–Whitney *U* test was applied to determine statistical significance (*p* < 0.05) at 95% confidence interval for numerical variables, that is, mean levels of anti-TPO, anti-TG, TSH, and free T4 between males and females, keeping in view skewed distribution of thyroid biomarkers. And the same test was applied for determining statistical significance between anti-TPO and anti-TG positive and negative groups versus TSH and free T4 levels. Odds ratio was calculated using online MedCalc calculator.

## 3. Results

Over a course of a ten-month study period, we received a total of 316 serum samples for anti-TPO/TG antibodies along with thyroid profile testing (TSH). These included 115 males (36.4%) and 201 females (63.6%). Their age ranged from 3 to 89 years (mean ± SD, 42.22 ± 18.09).

Table 1 shows comparison of prevalence and mean ranks for analyzed variables (anti-TPO antibodies, anti-TG antibodies, TSH, and free T4) among males and females. Anti-TPO antibodies are clearly found in statistically significant higher number of females and in higher levels. Anti-TG antibodies and TSH are found in more females and in higher levels but this difference did not achieve statistical significance.

In total 205 (65%) patients had normal TSH with mean ± SD 1.9 ± 1.42, while 111 (35%) had deranged TSH values, with mean ± SD 12.15 ± 18.96. Among first (normal TSH) group, mean rank of anti-TPO antibodies was 137.76, while, in second group (deranged TSH), mean rank of anti-TPO antibodies was 196.81, with *p* value 0.001 (and RR 2.3) through Mann–Whitney *U* test. Similarly, among first group, mean rank of anti-TG antibodies was 38.02 while in second group mean rank of anti-TG antibodies was 40.96, achieving *p* value of 0.098 (RR 2.12).

In total 107 (76%) patients had normal free T4 with mean ± SD 16.22 ± 3.08, while 34 (24%) had deranged free T4 values, with mean ± SD 31.53 ± 21.23. Among first (normal free T4) group, mean rank of anti-TPO antibodies was 68.93, while, in second group (deranged free T4), mean rank of anti-TPO antibodies was 77.5, with *p* value 0.286 (and RR 1.7) through Mann–Whitney *U* test. Similarly, among first group, mean rank of anti-TG antibodies was 20.76, while, in second group, mean rank of anti-TG antibodies was 19.96, achieving *p* value of 0.84 (RR 1.17).

Table 2 shows comparison of mean ranks of anti-TPO and anti-TG antibodies between TSH and free T4 normal and deranged groups. It indicates statistically significant difference among TSH normal and deranged groups when compared with anti-TPO antibodies as dependent variable. Odds ratio has been shown against each comparison.

## 4. Discussion

Anti-thyroid antibodies have long been known to affect thyroid function and influence thyroid profile testing. We had hypothesized that anti-thyroid antibodies are more often positive in individuals with deranged thyroid profile. In that we demonstrated that anti-TPO antibodies are more often present in individuals with deranged TSH as compared to those with normal TSH (*p* value 0.001, OR 4.3). This was not the case of anti-TPO antibodies with free T4 groups (*p* value 0.286, OR 2.0). Anti-TG antibodies are present in either TSH (normal versus deranged, *p* value 0.098, OR 4.2) or free T4 (normal versus deranged, *p* value 0.84, RR 1.27) groups at levels that did not attain statistical significance. Al Rabia has determined similar findings that TSH is more often deranged if anti-TPO (*p* value 0.036) or anti-TG (*p* value 0.000) antibodies are positive in Saudi population [15]. Similarly Brown et al. determined that anti-TPO positivity is associated with 60% increase in TSH (*p* value <0.001) [16]. They did not measure anti-TG antibodies. Loh et al. showed that TSH (and not free T4) is deranged more often in pregnancy if anti-TPO antibodies are positive (*p* value 0.04) as compared to if anti-TPO antibodies are negative [17]. In Iran, similar findings have been observed by Ghoraishian et al. They studied relationship of anti-TPO with T3, T4, and TSH in 2425 individuals and found these to be significantly deranged in antibody positive group [18]. The difference of their finding of free T4 (which we found insignificant) could be due to difference in sample size.

In addition to finding significant presence of anti-TPO antibodies in TSH deranged individuals, we also found that anti-TPO antibodies are more often raised in females as compared to males (*p* value 0.001) and their mean ranks are also higher (*p* value 0.002). Such statistically significant findings were not observed for anti-TG antibodies, TSH, or free T4 although their mean ranks were most often higher/abnormal in females in terms of percentages. Unnikrishnan et al. have reported higher prevalence of anti-TPO antibodies in females than in males (*p* < 0.05) in eight cities of India [19]. Hollowell et al. while conducting United States national health and nutrition examination survey determined that prevalence of both anti-TPO and anti-TG is higher in females (*p* < 0.001) [20]. Again this difference could be due to our smaller sample size for anti-TG antibodies. Similarly Dhakal et al. found higher prevalence of anti-TPO antibodies in females versus males (74% versus 26%) [21].

Although we have proved that anti-TPO antibodies are more often present in individuals with deranged TSH, it is difficult to establish a direct correlation between levels of these biomarkers. Anti-thyroid autoantibodies primarily exert their pathogenic effect through complement-dependent cytotoxicity and the relationship between presence of autoantibodies and development of future thyroid disease is complex [22]. Our inability to prove TSH versus anti-TG antibodies association or free T4 versus both antibodies association might be due to smaller sample size.

## 5. Conclusion

Anti-thyroid antibodies (specifically anti-TPO antibodies) are more often present when TSH is deranged. The individuals with deranged thyroid profile (specifically TSH) should also be screened for anti-thyroid antibodies to rule out underlying autoimmune phenomenon. This importance of screening is compounded by the fact that anti-thyroid antibodies may be positive in a significant percentage of elderly people.

## Figures and Tables

**Table 1:** Comparison of prevalence (Chi square test) and mean ranks (Mann–Whitney *U* test) for analyzed variables (anti-TPO antibodies, anti-TG antibodies, TSH, and free T4) among males and females.

	Males	Females	*p* value
Anti-TPO antibodies (*n* = 316)			
Prevalence% (positive/total)	22 (21/115)	37.8 (76/201)	**0.001**
Mean rank	137.66	170.43	**0.002**
Median + IQR	6 + 12	11 + 177	
Anti-TG antibodies (*n* = 82)			
Prevalence% (positive/total)	11 (3/26)	30 (17/56)	0.065
Mean rank	35.56	44.26	0.124
Median + IQR	25 + 47	39.5 + 185	
TSH (*n* = 316)			
Prevalence% (positive/total)	34 (39/115)	35.8 (72/201)	0.732
Mean rank	143.89	166.86	0.031
Median + IQR	1.5 + 2.39	2.1 + 3.55	
Free T4 (*n* = 141)			
Prevalence% (positive/total)	23.4 (11/47)	24.4 (23/94)	0.59
Mean rank	70.94	71.03	0.99
Median + IQR	17.4 + 6.9	17.6 + 6.1	

**Table 2:** Comparison of mean ranks of anti-TPO and anti-TG antibodies between TSH and free T4 normal and deranged groups.

	TSH				Free T4			
	Normal TSH (0.4–4.5 mIU/ml)	Deranged TSH (<0.4 or >4.5)	*p* value	OR (95% CI)	Normal T4 (8–21 pmol/l)	Deranged T4 (<8 or >21)	*p* value	OR (95% CI)
Anti-TPO antibodies mean rank	137.76 (*n* = 205)	196.81 (*n* = 111)	**0.001**	4.3 (2.62–7.23)	68.93 (*n* = 107)	77.5 (*n* = 34)	0.286	2.0 (0.93–4.45)
Anti-TG antibodies mean rank	38.02 (*n* = 50)	40.96 (*n* = 32)	0.098	4.2 (1.44–12.2)	20.76 (*n* = 27)	19.96 (*n* = 13)	0.840	1.27 (0.29–5.46)

## References

[1] Stathatos N., Daniels G. H. (2012). Autoimmune thyroid disease. *Current Opinion in Rheumatology*.

[2] Antonelli A., Ferrari S. M., Corrado A., Di Domenicantonio A., Fallahi P. (2015). Autoimmune thyroid disorders. *Autoimmunity Reviews*.

[3] Iddah M. A., Macharia B. N. (2013). Autoimmune thyroid disorders. *ISRN Endocrinology*.

[4] Aruna N., Pushpa N. (2015). Pregnancy outcome in euthyroid women with anti thyroid peroxidase antibodies. *The Journal of Obstetrics and Gynecology of India*.

[5] Walsh J. P., Bremner A. P., Feddema P., Leedman P. J., Brown S. J., O'Leary P. (2010). Thyrotropin and thyroid antibodies as predictors of hypothyroidism: a 13-year, longitudinal study of a community-based cohort using current immunoassay techniques. *The Journal of Clinical Endocrinology & Metabolism*.

[6] Roos A., Links T. P., De Jong-Van Den Berg L. T. W., Gans R. O. B., Wolffenbuttel B. H. R., Bakker S. J. L. (2010). Thyroid peroxidase antibodies, levels of thyroid stimulating hormone and development of hypothyroidism in euthyroid subjects. *European Journal of Internal Medicine*.

[7] Mikoś H., Mikoś M., Obara-Moszyńska M., Niedziela M. (2014). The role of the immune system and cytokines involved in the pathogenesis of autoimmune thyroid disease (AITD). *Endokrynologia Polska*.

[8] Hutfless S. M., Matos P., Talor M. V., Caturegli P., Rose N. R. (2011). Significance of prediagnostic thyroid antibodies in women with autoimmune thyroid disease. *The Journal of Clinical Endocrinology & Metabolism*.

[9] McLeod D. S. A., Cooper D. S. (2012). The incidence and prevalence of thyroid autoimmunity. *Endocrine Journal*.

[10] Rose N. R. (2007). Prediction and prevention of autoimmune disease: a personal perspective. *Annals of the New York Academy of Sciences*.

[11] Khan A., Khan M. M. A., Akhtar S. (2002). Thyroid disorders, etiology and prevalence. *Journal of Medical Sciences*.

[12] Riaz M., Afzal N., Mahmud T. H. (2015). The high percentages of anti thyroid antibodies positive SLE patients at Sheikh Zayed Hospital, Lahore (Pakistan). *Majmaah Journal of Health Sciences*.

[13] Shafiq M. I., Gauhar A., Akram M., Elahi S. (2015). Thyroid peroxidase antibodies in non-interferon treated hepatitis C patients in Pakistan. *BioMed Research International*.

[14] Aamir I. S., Tauheed S., Majid F., Atif A. (2010). Frequency of autoimmune thyroid disease in chronic urticarial. *Journal of the College of Physicians and Surgeons Pakistan*.

[15] Al-Rabi M. W. (2017). Correlation of thyroid antibodies with TSH, T3 and T4 hormones in patients diagnosed with autoimmune thyroid disorders. *Pakistan Journal of Pharmaceutical Sciences*.

[16] Brown S. J., Bremner A. P., Hadlow N. C. (2016). The log TSH–free T4 relationship in a community-based cohort is nonlinear and is influenced by age, smoking and thyroid peroxidase antibody status. *Clinical Endocrinology*.

[17] Loh T. P., Tee J. C. S., Tee N. W. S. (2016). Association between thyroid function tests and anti-thyroid peroxidase (TPO) antibodies in pregnancy. *Endocrine Journal*.

[18] Ghoraishian S. M., Hekmati Moghaddam S. H., Afkhami-Ardekani M. (2006). Relationship between anti-thyroid peroxidase antibody and thyroid function test. *Iranian Journal of Immunology*.

[19] Unnikrishnan A. G., Kalra S., Sahay R. K. (2013). Prevalence of hypothyroidism in adults: an epidemiological study in eight cities of India. *Indian Journal of Endocrinology and Metabolism*.

[20] Hollowell J. G., Staehling N. W., Flanders W. D. (2002). Serum TSH, T4, and Thyroid Antibodies in the United States Population (1988 to 1994): National Health and Nutrition Examination Survey (NHANES III). *The Journal of Clinical Endocrinology & Metabolism*.

[21] Dhakal S., Nagila A., Koirala R., Bhatta M. P., Regmi S., Hamza A. (2017). Correlation of anti-thyroid peroxidase antibodies (anti-TPO) with thyroid hormones in local population of Western Nepal. *International Journal of Advanced Research*.

[22] McLachlan S. M., Rapoport B. (2007). Thyroid peroxidase as an autoantigen. *Thyroid*.

